# Anticancer Drug Development: Preclinical Screening, Clinical Trials and Approval

**DOI:** 10.1038/sj.bjc.6602070

**Published:** 2004-08-24

**Authors:** D Selwood

**Affiliations:** 1 Wolfson Institute for Biomedical Research, University College London, UK

This book provides a very comprehensive overview of *in vitro* and *in vivo* screening methodologies and toxicology, together with clinical trials design. As a medicinal chemist who has encountered many of the *in vitro* assays and some of the *in vivo* assays described, this volume provides a fascinating rare, global view of this vast subject.

The book is divided into four sections: *in vitro* methods, *in vivo* methods, nonclinical testing (toxicology) and clinical testing. Separate chapters with mostly different authors contribute to each chapter. A welcome feature is the supply of an e-book with the printed volume, especially useful given the size and weight of the conventional volume. Two IT personnel, 1 h, a new copy of Acrobat reader 6 and the e-book sat easy to access on my laptop. Simple search functions are available in this program, but no Boolean logic with the free version. The general presentation of the book is poor, with the figures probably published as presented by the authors. There is no use of colour at all, sorely missed in those sections including histology. There is no glossary, a key omission if the book is intended for a wider audience. Fortunately, there is an index. The book has the feel of a conference proceedings rather than a quality textbook or monograph.

These misgivings aside, the volume is bursting with useful information for the career professional and the interested scientist. For such a comprehensive treatise, I can only select a few highlights. Chapter 2 on high-throughput screening contributed by Bioclair and colleagues is an easy-to-follow guide to the latest state of the art in this area. Chapter 6 on the use of solid tumour model in rodents for tumour screening by Corbett and colleagues was for me the highlight of the book, providing an exceptionally clear and well-written guide to this vital area. The examples on how to conduct an *in vivo* assay (and more to the point how not to) should be required for reading for professionals in this area. However, there are many other highlights: Chapter 9 on orthotopic metastatic models, Chapter 12 on the use of companion animals for tumour screening is an eye opener. The vast number of such animals and their caring owners will likely provide a useful resource for evaluation of novel therapies in the future.

In the pre-clinical and clinical trials sections, the book switches from a review format to be more of a professional guide. Detailed descriptions of NDA and NCI requirements and panels are provided and these will prove useful to the specialist. There is also a chapter on the different scenario for clinical testing in Europe. I found Section III on nonclinical testing (toxicological testing) the best organised of the book. Perhaps this is to be expected as it was written by a small number of authors (Roy, Andrews and Laurencot). These authors cover the different aspects of planning and conducting toxicology studies for both small molecules and biologics. The authors make a key point with relevance to clinical testing of a combination of drugs, namely that the potential adverse synergistic effects must be considered in addition to the potential benefits.

The one aspect I missed overall was a forward-looking chapter from each section. What are the key problems with *in vitro* and *in vivo* methods? What avenues are being explored to overcome these problems? Also are biologics dealt with in sufficient detail in the book, what special problems do they present for the development process? These gripes aside, I feel I will be consulting this volume for years to come when I require information about a specific aspect of the anti-cancer drug development process.

