# Anticancer drug development: preclinical screening, clinical trials and approval

**DOI:** 10.1038/sj.bjc.6602286

**Published:** 2004-11-23

**Authors:** B A Teicher, D L Selwood, P A Andrews

**Correction to:** *British Journal of Cancer* (2004) **91**, 1000. doi:10.1038/sj.bjc.6602070

Due to an error, the ISBN number of the book reviewed above was given incorrectly. The correct number is shown below:

ISBN – 1588292282.

